# Machine learning models predicting undertriage in telephone triage

**DOI:** 10.1080/07853890.2022.2136402

**Published:** 2022-10-26

**Authors:** Ryota Inokuchi, Masao Iwagami, Yu Sun, Ayaka Sakamoto, Nanako Tamiya

**Affiliations:** a Department of Health Services Research, Faculty of Medicine, University of Tsukuba, Tsukuba, Japan; b Health Services Research and Development Center, University of Tsukuba, Tsukuba, Japan; c Graduate School of Comprehensive Human Sciences, University of Tsukuba, Tsukuba, Japan

**Keywords:** Prehospital, after-hours house-call medical service, out-of-hour service, prediction

## Abstract

**Background:**

Undertriaged patients have worse outcomes than appropriately triaged patients. Machine learning provides better triage prediction than conventional triage in emergency departments, but no machine learning-based undertriage prediction models have yet been developed for prehospital telephone triage. We developed and validated machine learning models for telephone triage.

**Materials and methods:**

We conducted a retrospective cohort study with the largest after-hour house-call (AHHC) service dataset in Japan. Participants were ≥16 years and used the AHHC service between 1 November 2018 and 31 January 2021. We developed five prediction models based on support vector machine (SVM), lasso regression (LR), random forest (RF), gradient-boosted decision tree (XGB), and deep neural network (DNN). The primary outcome was undertriage, and predictors were telephone triage level and routinely available telephone-based data, including age, sex, 80 chief complaint categories and 10 comorbidities. We measured the area under the receiver operating characteristic curve (AUROC) for all the models.

**Results:**

We identified 15,442 eligible patients (age: 38.4 ± 16.6, male: 57.2%), including 298 (1.9%; age: 58.2 ± 23.9, male: 55.0%) undertriaged patients. RF and XGB outperformed the other models, with the AUROC values (95% confidence interval; 95% CI) of the SVM, LR, RF, XGB and DNN for undertriage being 0.62 (0.55–0.69), 0.79 (0.74–0.83), 0.81 (0.76–0.86), 0.80 (0.75–0.84) and 0.77 (0.73–0.82), respectively.

**Conclusions:**

We found that RF and XGB outperformed other models. Our findings suggest that machine learning models can facilitate the early detection of undertriage and early intervention to yield substantially improved patient outcomes.

**KEY MESSAGES**
- Undertriaged patients experience worse outcomes than appropriately triaged patients; thus, we developed machine learning models for predicting undertriage in the prehospital setting. In addition, we identified the predictors of risk factors associated with undertriage.
- Random forest and gradient-boosted decision tree models demonstrated better prediction performance, and the models identified the risk factors associated with undertriage.
- Machine learning models aid in the early detection of undertriage, leading to significantly improved patient outcomes and identifying undertriage-associated risk factors, including chief complaint categories, could help prioritize conventional telephone triage protocol revision.

## Introduction

Undertriage has been defined as the proportion of patients who are allocated to a lower urgency category than the reference category [1], leading to worsened prognosis due to delays in therapeutic intervention. In prehospital telephone triage, a previous systematic review and clinical trial showed that undertriage resulting from the use of telephone triage protocols varies from 0.04% to 10% [2,3], and to decrease undertriage, several countries have developed and revised their telephone triage protocols [4]. Similarly, in emergency departments, undertriaged patients experience worse outcomes than appropriately triaged patients [5,6]; thus, to minimize undertriage, countries have revised their emergency department triage protocols.

Recently, machine learning models have been developed and have shown better triage prediction performance than the conventional triage methods for chief complaint categories and vital signs usually observed in emergency departments [7–10]. However, there are no reports of machine learning models predicting undertriage in prehospital telephone triage.

Alerting patients who have a high likelihood of undertriage can facilitate the early detection of undertriage and early intervention and yield substantially improved patient outcomes. Thus, to predict undertriage based on information obtained by telephone, we developed five machine learning models based on the support vector machine (SVM), lasso regression (LR), random forest (RF), gradient-boosted decision tree (XGB) and deep neural network (DNN) models. In addition, we identified the predictors of risk factors associated with undertriage.

## Materials and methods

### Data source

This study used anonymized data from the medical records of patients who used the after-hours house-call (AHHC) service provided by Fast DOCTOR Ltd. (Tokyo, Japan). The database used in this study is private and includes the following variables (1) before and (2) after consultation: (1) patient age, sex, chief complaint categories, comorbidities (hypertension, diabetes mellitus, hyperlipidaemia, gout, chronic lung disease, heart failure, liver disease, cerebral infarction, cancer and dementia), and telephone triage level and (2) doctor’s triage level.

We followed the reporting guidelines from the Transparent Reporting of a multivariable prediction model for Individual Prognosis or Diagnosis statement [9]. The study design was reviewed and approved by the Research Ethics Committee of the University of Tsukuba (approval number: 1527). The need for written informed consent from patients for publication of their details was waived.

### AHHC medical service

Several developed countries provide AHHC medical services that deploy doctors directly to patients’ residences [11,12]. In Japan, a large private AHHC service operates seven days per week outside regular hospital hours (18:00–06:00 from Monday to Saturday and 24 h on Sundays and holidays). We previously reported on the private AHHC service in Japan [13–15]. In brief, when a patient calls the AHHC service, the operators (trained telephone triage nurses) perform telephone triage according to the well-used telephone triage protocol developed by the Fire and Disaster Management Agency, and the operator then classifies the patient into one of the following five categories: “need immediate hospital visit by ambulance (red),” “need to visit a hospital within 1 h (orange),” “need to visit a hospital within 6 h (yellow),” “need to visit a hospital within 24 h (green)” and “do not need a hospital visit (white).”

The AHHC service sends a doctor when a patient is triaged as “orange“ or “yellow.” If the patient is triaged as “red,” the AHHC service calls an ambulance. If the patient is triaged as either “green” or “white,” they are guided to a nearby clinic or hospital. After this service, the doctor performs a home visit for the patients and classifies the patients into three categories: grade 1 (can be treated using over-the-counter medications), grade 2 (requiring a hospital or clinic visit) or grade 3 (requiring ambulance transportation).

### Definition of undertriage

We defined patients who were initially classified as “orange“ or “yellow“ in the telephone triage but were subsequently triaged as grade 3 by the AHHC doctor as undertriaged.

### Participants

We included all patients aged ≥16 years who used the AHHC service from 1 November 2018 to 31 January 2021. We excluded patients who were aged <16 years, those not categorized in the yellow or orange categories, and those for whom there was no chief complaint category.

### Outcome and predictors

The primary outcome was undertriage. As predictors for the machine learning models, we included routinely available information from the telephone triage settings: age, sex, chief complaint categories, comorbidities and telephone triage level.

### Missing data

We performed imputation to account for missing data with regard to age, sex, telephone triage level, and doctor’s assessment using the *k*-nearest neighbours algorithm [16]. The following covariates were used for the imputation: age, sex, chief complaint, comorbidities and telephone triage level. The unobserved data for binary variables of comorbidities were set to zero in all the models.

### Statistical analysis

#### Development of machine learning models

First, the dataset was randomly divided into two subsets: 70% of the patients were included in the training set and 30% were included in the test set [17]. Second, one-hot encoding, wherein a new binary feature is created for each possible category and a value of one is assigned to the feature of each sample that corresponds to its original category, was performed at the chief complaint category level in both the sets. Third, we standardized the feature variables with a mean of zero and a standard deviation (SD) of one. Fourth, using the training set, we developed models based on the SVM, LR, RF, XGB and DNN for each outcome using the predictors. LR can effectively exclude predictors from the final model by shrinking their coefficients to exactly zero [18,19]. SVM aims to find the hyperplane with a maximal distance between pairs of classes [20]. RF models comprise ensembles of decision trees constructed from bootstrapped training samples for which random samples corresponding to specific numbers of predictors are selected to initiate tree induction [21]. XGB is another ensemble method in which new tree models for predicting the errors and residuals of previous models are constructed. When these new models are combined, XGB uses a gradient descent algorithm to minimize the loss function [22]. DNN models comprise multiple processing layers and model outcomes *via* intermediate hidden units, each comprising a linear combination of predictors that are transformed into nonlinear functions [20].

To minimize potential overfitting, 10-fold cross-validation was performed with all the models on the training dataset, and all dummy variables corresponding to rare categories, for which the cut-off was 10 variables, were removed [23]. Fifth, we performed hyperparameter optimisation on the training set to improve the performance of each model [24]. Specifically, we attempted to use six random hyperparameter values (tuneLength = 6; the *caret* package in R) in the SVM, and automated machine learning (the *h2o* package in R) in RF, XGB, and DNN (Supplemental Table S1). Finally, we used the top 20 variables with high importance to examine the contribution of each predictor to the model with the best discriminatory abilities. All measures of variable importance were scaled to have a maximum value of 100.

#### Validation of the developed machine learning models

To evaluate the performance of each model on the test set, we calculated the area under the receiver operating characteristic curve (AUROC) and used a confusion matrix to summarize the results (sensitivity [recall], specificity, positive predictive value [precision] and negative predictive value). To compare the AUROC values between each model, we used Delong’s test [25].

#### Sensitivity analysis

First, as a complete case analysis, we repeated the analyses using complete cases for age, sex, telephone triage level, and doctor grade assessments after consultations. Second, because the incidence proportion of the primary outcome was small (as shown later), we used the Synthetic Minority Over-sampling Technique for Nominal and Continuous (SMOTE-NC) [26] algorithm in the training set. SMOTE-NC uses the k-nearest neighbours algorithm, and we set *k* = 5. We also applied under-sampling (ratio of the number of undertriaged to non-undertriaged cases = 1:3). Finally, we performed min-max normalisation to ages between zero and one for the training and test sets.

#### Libraries for data analyses and machine learning

The baseline characteristics, measured as continuous or categorical variables, were summarized. The categorical data were expressed as percentages. The normally and non-normally distributed variables were expressed in the form of mean (SD) and median (interquartile range), respectively. The chi-square test was used to compare categorical data; however, when the expected cell counts were five or fewer, the Fisher’s exact test was used. The continuous variables were compared using Welch’s *t*-test or the Mann–Whitney U test, depending on the data distribution. A two-sided *p* < .05 was considered statistically significant. The data analysis was performed using R version 4.1.2, (R Foundation for Statistical Computing, Vienna, Austria) with the *RSBID* package for SMOTE-NC; *caret* package for SVM; and *h2o* package for LR, RF, XGB and DNN.

### Role of the funding source

The funder gathered the source data but did not participate in the analysis, interpretation and/or writing of this manuscript. All authors had access to the raw dataset.

## Results

### Patient characteristics

**Figure 1.**
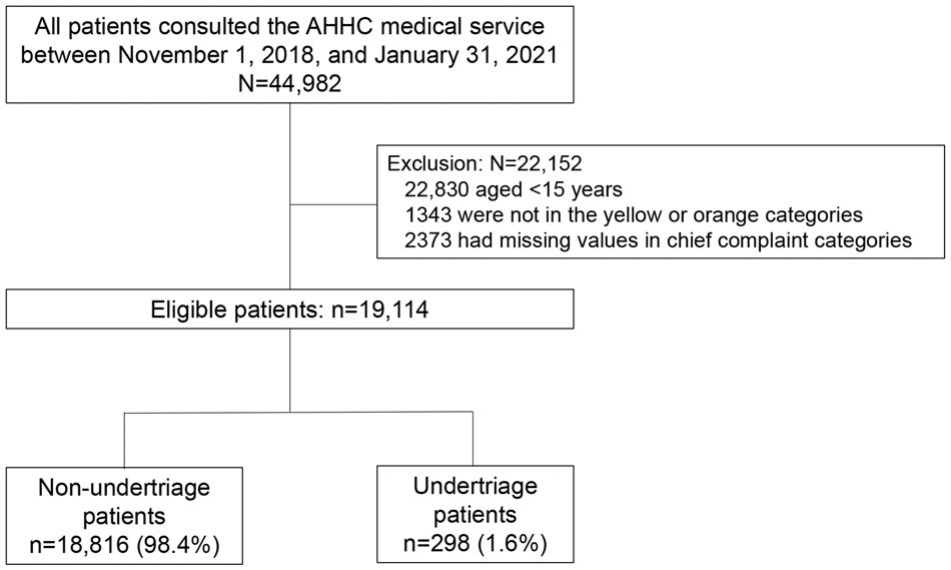
Flow diagram of patient selection for model development.

A total of 44,982 patients consulted the AHHC service between 1 November 2018 and 31 January 2021. We excluded 22,152 patients, including those aged under 15 years, not in the yellow or orange categories, and lacking chief complaint categories. A total of 19,114 patients were eligible, and the primary outcome of undertriage was 298 patients (1.9%; Figure 1). Missing data included telephone triage levels and doctor assessments, 3899 (20.4%) and 363 (1.9%), respectively (Table 1 and Supplemental Table S2).

**Table 1.** Patient characteristics.

Characteristic	Total (*n* = 19,114)
Age, mean ± (*SD*)	38.4 (16.6)
Category, *n* (%)	
15–64	17,364 (90.8)
65–74	643 (3.4)
>75	1107 (5.8)
Male, *n* (%)	10,915 (57.2)
Missing	38 (0.3)
Triage colour	
Yellow	8567 (30.7)
Orange	10,547 (69.3)
Missing	3899 (20.4)
Doctor assessment	
Grade 1	9278 (49.5)
Grade 2	9133 (48.7)
Grade 3	343 (1.8)
Missing	363 (1.9)
Undertriage, *n* (%)	298 (1.6)
Missing	3918 (20.5)
Comorbidities, *n* (%)	
Hypertension	780 (4.1)
Diabetes mellitus	341 (1.8)
Cancer	460 (2.4)
Chronic lung disease	723 (3.8)
Myocardial infarction	48 (0.3)
Heart failure	32 (0.2)
Cerebral infarction	136 (0.7)
Immun*e* = 1 (%)	30 (0.2)
Hyperlipidaemia	238 (1.2)
Liver disease	87 (0.5)
Dementia	91 (0.5)
Gout	81 (0.4)
Telephone triage symptom, *n* (%)	
Common cold symptoms	8900 (46.6)
Syncope	2049 (10.7)
Sore throat	1383 (7.2)
Headache	1150 (6.0)
Diarrhoea	856 (4.5)
Rash	498 (2.6)
Constipation	434 (2.3)
Allergic reaction	353 (1.8)
Fever (adult)	290 (1.5)
Bruising	266 (1.4)
Ear ringing	255 (1.3)
Ankle to toe problem	249 (1.3)
Lower extremity problem	195 (1.0)
Blood in stool	189 (1.0)
Laceration	184 (1.0)
Dyspnoea	130 (0.7)
Upper extremity problem	129 (0.7)
Burn	119 (0.6)
Abdominal pain	117 (0.6)
Chest pain	108 (0.6)
Asthma	89 (0.5)
Bleeding	82 (0.4)
Back pain	82 (0.4)
Genital problems in male	72 (0.4)
Dizziness	71 (0.4)
Extremity/facial injury	70 (0.4)
Itching	65 (0.3)
Wheezing	57 (0.3)
Mastalgia	54 (0.3)
Pain during urination	47 (0.2)
Numbness, sensory disturbance, paralysis	45 (0.2)
Hiccups	45 (0.2)
Palpitation	43 (0.2)
Abnormal urine colour	38 (0.2)
Fall	38 (0.2)
Dysuria	34 (0.2)
Bites animal/human/insect/marine animal/snake	32 (0.2)
Anxiety or fear	30 (0.2)
Heat stroke	30 (0.2)
Neck pain	28 (0.1)
Hyperventilation	25 (0.1)
Wound healing and infection	24 (0.1)
Head injury	17 (0.1)
Vomiting or nausea	14 (0.1)
Neck injury	14 (0.1)
Heartburn	13 (0.1)
Hypertension	12 (0.1)
Disturbance of consciousness	12 (0.1)
Nasal injury	11 (0.1)
Earache, drainage	8 (0.0)
Eye injury	8 (0.0)
Hearing loss	6 (0.0)
Food poisoning	5 (0.0)
Vaginal bleeding	4 (0.0)
Penetrating injury	4 (0.0)
Trunk injury	4 (0.0)
Contact lens problems	3 (0.0)
Dysarthria	2 (0.0)
Lumbago	2 (0.0)
Eye problems	2 (0.0)
Nasal problem	2 (0.0)
Seizure	2 (0.0)
Foreign body, eye	2 (0.0)
Foreign body, skin	2 (0.0)
Insomnia	1 (0.0)
Depression	1 (0.0)
First aid for trauma and burns	1 (0.0)
Swallowing of foreign body	1 (0.0)
Accidental ingestion of liquids	1 (0.0)
Foreign body, nose	1 (0.0)
Fish bone in the pharynx	1 (0.0)
Foreign body, rectum	1 (0.0)
Foreign body, vagina	1 (0.0)
Hypothermia	1 (0.0)

SD: standard deviation.

Among the 19,114 patients, the mean patient age was 38.4 ± 16.6 years, and 57.2% were male. Major comorbidities were hypertension and chronic lung disease (4.1% and 3.8%, respectively), and the chief complaints were common cold symptoms and syncope (46.6% and 10.7%, respectively).

Meanwhile, among the 298 patients with undertriage, the mean age was 58.2 ± 23.9 years, and 55.0% were male. Major comorbidities were hypertension, cancer and diabetes mellitus (14.4%, 8.4% and 7.7%, respectively), and the chief complaints were common cold symptoms, sore throat and syncope (20.5%, 13.1% and 11.4%, respectively; Supplemental Table S3).

#### Predicting primary outcome:

Of the 19,114 patients, 13,379 (70%) and 5735 (30%) were allocated to the training and test datasets, respectively. The candidate predictors and primary outcome did not differ between the training and test sets (Supplemental Table S4).

**Figure 2.**
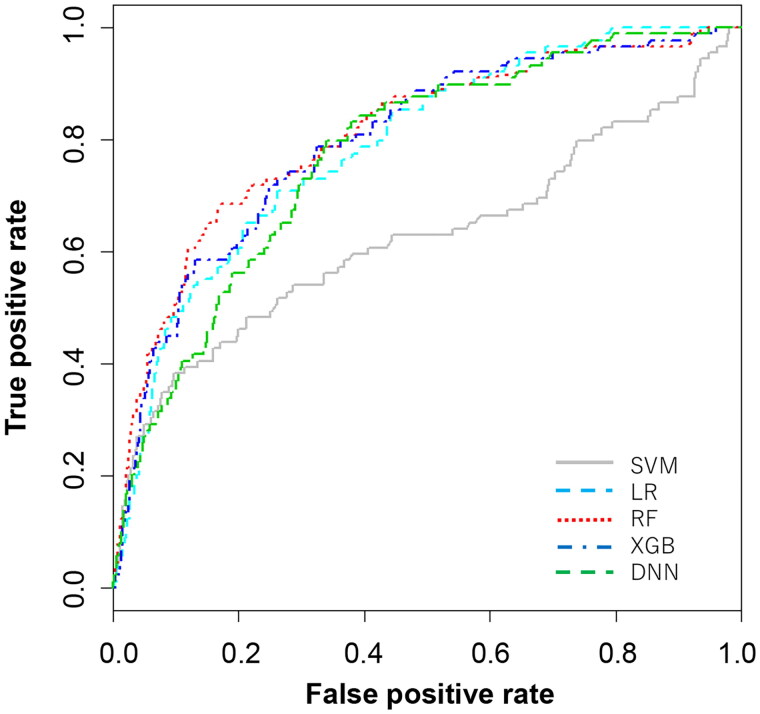
AUROC evaluated in the test set. The best cut-off point was set as the cut-off value to the point on the AUROC curve that maximizes the sum of sensitivity + specificity – 1, that is, the Youden index that provides an efficient trade-off between sensitivity and specificity. AUROC: area under the receiver operating characteristic curve; DNN: deep neural network; LR: lasso regression; RF: random forest; SVM: support vector machine; XGB: extreme gradient boosting.

The discriminatory abilities of the five models are presented in Table 2 and Figure 2. The RF model demonstrated good discrimination ability; the AUROCs (95% CI) were as follows: SVM, 0.62 (0.55–0.69); LR, 0.79 (0.74–0.83); RF, 0.81 (0.76–0.86); XGB, 0.80 (0.75–0.84); and DNN, 0.77 (0.73–0.82) (Delong test *p* < .01). The RF, XGB, and DNN models had high sensitivity (72.0%, 72.0% and 79.8%, respectively). All the models had high negative predictive values owing to the low prevalence of the undertriage.

**Table 2.** Discriminatory abilities of the machine learning models.

Classifiers	Sensitivity	Specificity	PPV	NPV	AUROC (95% CI)
SVM	53.9	71.4	2.9	99.0	0.62 (0.55–0.70)
LR	70.8	73.8	4.1	99.4	0.79 (0.74–0.83)
RF	71.9	77.9	4.9	99.4	0.81 (0.76–0.86)
Gradient-boosted decision tree	71.9	75.0	4.3	99.4	0.80 (0.75–0.84)
DNN	79.8	66.1	3.6	99.5	0.77 (0.73–0.82)

AUROC: area under the receiver operating characteristic curve; CI: confidence interval; NPV: negative predictive value; PPV: positive predictive value.

### Top 20 permutation-based variable importance

**Figure 3.**
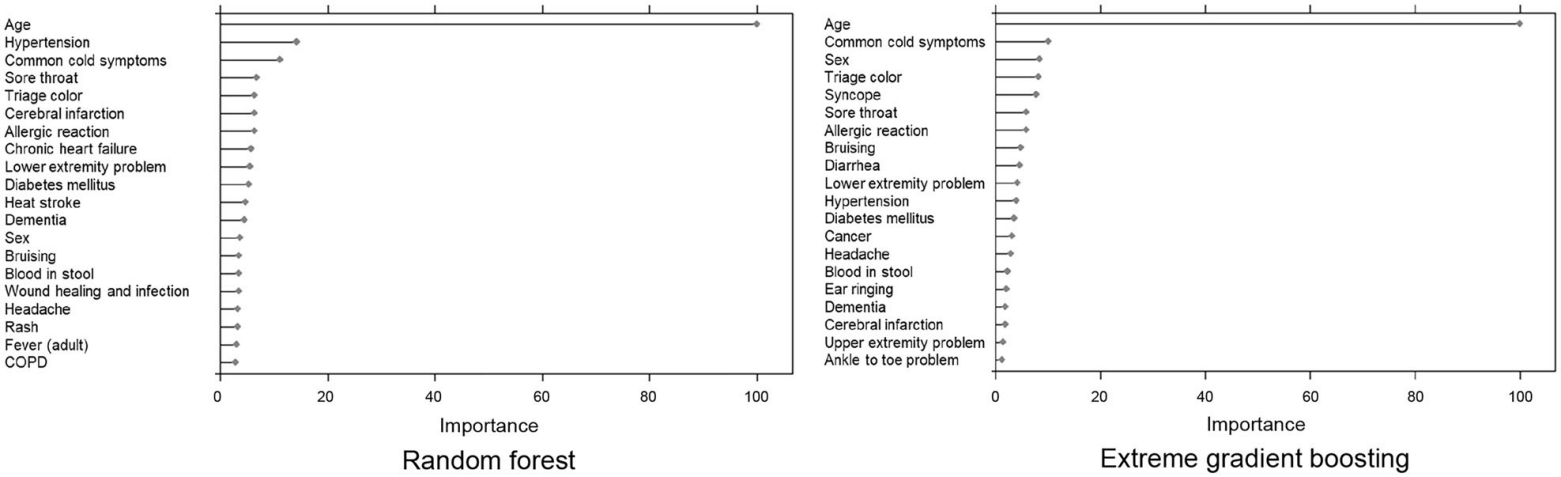
Top 20 most-important variables for the RF and extreme gradient boosting models.

Figure 3 shows the summary plots for the top 20 most important permutation-based variables for RF and XGB. The important variables were similar for both the models. Age, male, comorbidities (including hypertension, diabetes mellitus, cerebral infarction and dementia), and chief complaint categories (common cold symptom, sore throat, headache, allergic reaction, bruising, blood in stool and lower extremity problem) had high importance. In the case of LR, similar results were observed. (Supplemental Table S5).

### Sensitivity analysis

We analysed the complete case without multiple imputations, performed under- and over-sampling (SMOTE-NC), and min-max normalisation on age; this resulted in lower predictive performance than in our main analysis (Supplemental Tables S6–S8).

## Discussion

We developed and validated five machine learning models for undertriage prediction. Among the machine learning models tested, RF and XGB demonstrated better performance. Additionally, the developed models identified the risk factors in the telephone triage protocol associated with undertriage.

Performing accurate triage over the telephone can be extremely time-consuming; thus, telephone triage must be performed with the minimum and routinely available information through the telephone, which is helpful for both triage nurses and patients.

To the best of our knowledge, this is the first study to use machine learning for undertriage prediction. The use of machine learning models can aid in the early detection of undertriage, whereas telephone triage can facilitate intervention *via* early consultation, which can lead to significantly improved patient outcomes. Consequently, identifying the risk factors for undertriage in telephone triage categories can help prioritize the revision of telephone triage protocols.

### Machine learning models for predicting undertriage

In the present study, RF and XGB achieved better performances than DNN. Our dataset primarily comprised categorical data because the information obtained *via* telephone interviews mainly consisted of categorical variables, such as the gender, chief complaint, and symptoms; this is in contrast to a hospital setting wherein the data mainly consist of continuous variables, such as vital signs and laboratory data. Thus, the RF and XGB models may have high predictive performance in prehospital settings.

### Variable importance

Permutation-based variable importance showed that age and comorbidities (including hypertension, diabetes mellitus, cerebral infarction, and dementia) contribute to undertriage, which is consistent with the results of previous studies [27,28]. In addition, some chief complaint categories are highly crucial for undertriage. It is therefore crucial to prioritize future changes in the original source telephone triage protocol.

### Priority of the telephone triage protocol revision

The development of prehospital telephone triage protocols that can identify appropriate care required for patients and determine their need for immediate hospital visit by ambulance remains challenging. Protocol revision is time- and effort-intensive. However, our approach makes it possible to identify the factors that are associated with undertriage in several chief complaint categories in telephone triage protocols and is expected to help in the revision of protocols in various countries.

### Strengths and limitations

This is the first study to predict undertriage using machine learning algorithms. Machine learning can aid in the early detection of undertriage, whereas telephone triage can facilitate timely intervention *via* early consultation, leading to significantly improved patient outcomes. Additionally, insights into machine learning approaches enable the identification of factors associated with undertriage in several chief complaint categories in the telephone triage protocols that have low incidence. This can help prioritize protocol revision in various countries.

This study has several limitations. First, our prediction model is not generalisable for application at a global level because this study was performed based on a single AHHC service in Japan; thus, it may be difficult to adapt our results to other triage levels and countries. However, our approach is useful for application in other telephone triage protocols for evaluating factors associated with undertriage in other countries. Second, the study included a relatively small number of undertriage patients; thus, we used over- and under-sampling to avoid overfitting and performed several sensitivity analyses. There is no consensus on the definition of imbalanced data, but if the prevalence is under 1%, the SMOTE method may provide a higher predictive performance. Third, we imputed the missing values using the k-nearest neighbours algorithm. This method is simple and widely used for imputing missing data; however, it can generate bias. Therefore, we performed a sensitivity analysis to use the complete data and obtained similar results. Fourth, the composite outcome was obtained before the pandemic. As the COVID-19 pandemic affected telephone triage assessments, periodic revisions of the models are needed in the future. Fifth, we did not evaluate inter-rater reliability among the AHHC service doctors. Sixth, the AHHC service could not follow-up with the patients assessed in the undertriage group sent to the hospital. If very few patients sent to the hospital are admitted and most are sent home, it can be considered that the telephone triage protocols are working, and some physicians might assess the patient as overtriaged. Sixth, in this study, we did not follow-up with the patients classified as “green“ or “white,” leading to undertriage underestimation. It finally, the triage level may have changed by the time the AHHC doctor arrived; the reason for this change should be investigated in the future.

## Conclusion

This study developed undertriage prediction models using machine learning algorithms and identified the risk factors associated with undertriage. Our study provides insight into determining the potential of machine learning-based prediction models for undertriage. In the future, introducing machine learning models into telephone triage could facilitate the early detection of undertriage and potentially improve patient outcomes. Additionally, targeting the identified factors and chief complaint categories in telephone triage associated with undertriage can help in prioritising the revision of conventional telephone triage protocols.

## Supplementary Material

Supplemental Material

## Data Availability

The datasets used and/or analysed during the current study are available from the corresponding author, RI, upon reasonable request.
