# Supplementary material for: Machine learning models predicting undertriage in telephone triage
Source: Ann Med. 2022 Oct 26;54(1):2990–7. doi: 10.1080/07853890.2022.2136402 (PMC9621252; doi:10.1080/07853890.2022.2136402)
Supplement: Supplemental Material [file IANN_A_2136402_SM1179.docx]

**Appendix**

**APPENDIX**

**Supplemental Tables** 1

Table S1. Tuning of hyperparameters for each model 2

1) Hyperparameters for the support vector machine

2) Hyperparameters for the random forest

3) Hyperparameters for the extreme gradient boosting model

4) Hyperparameters for the neural network model

Table S2. Comparison of characteristics between patients with and without missing values　4

Table S3. Characteristics of patients with undertriage 7

Table S4. Comparison of patient characteristics between the training and test datasets 9

Table S5. Lasso logistic regression for the undertriage 10

Table S6. Comparison of patient characteristics on the training set before and after SMOTE-NC 12

Table S7. Comparison of patient characteristics on the training set after SMOTE-NC and test set 　　　　　　　　 15

Table S8. Results of sensitivity analyses. 18

1) Complete case analysis

2) Under-sampling method

3) Over-sampling method

4) Min-max normalization to age

Table S9. TRIPOD Checklist 19

## Table S1. Tuning of hyperparameters for each modeling algorithm

1) Hyperparameters for the support vector machine

| Name | Best parameters |
| --- | --- |
| C | 1 |
| gamma | 0.12 |

### 2) Hyperparameters for the random forest model

| Name | Best parameters |
| --- | --- |
| number of trees | 35 |
| number of internal trees | 35 |
| model size in bytes | 60,992 |
| min depth | 20 |
| max depth | 20 |
| mean depth | 20 |
| min leaves | 79 |
| max leaves | 205 |
| mean leaves | 132.9 |

### 3) Hyperparameters for the extreme gradient boosting model

| Name | Best parameters |
| --- | --- |
| col sample rate | 0.8 |
| col sample rate per tree | 0.8 |
| fold assignment | Modulo |
| number of trees | 26 |
| number of internal_trees | 26 |
| model size in bytes | 19,042 |
| min depth | 11 |
| max depth | 15 |
| mean depth | 14.6 |
| min leaves | 23 |
| max leaves | 74 |
| mean leaves | 53.2 |
| min rows | 100 |
| stopping tolerance | 0.009 |
| histogram type | UniformAdaptive |

### 4) Hyperparameters for the neural network model

| Name | Best parameters |
| --- | --- |
| epochs | 10.8 |
| layer | 5 |
| layer 1 units | 142 |
| layer 2 units | 10 |
| layer 3 units | 10 |
| layer 4 units | 10 |
| layer 5 units | 2 |

Table S2. Comparison of characteristics between patients with and without missing values

|  | without missing (n = 15,204) | missing (n = 3,910) | *p-value* |
| --- | --- | --- | --- |
| Age mean ± SD | 39.2 ± 17.3 | 35.3 ± 12.9 | <0.001 |
| Male (%) | 8629 (56.8) | 2286 (59.1) | 0.009 |
| Triage color |  |  | 0.364 |
| Yellow | 4664 (30.7) | 6 (46.2) |  |
| Orange | 10540 (69.3) | 7 (53.8) |  |
| Undertriage | 298 (2.0) | 0 (0.0) | > 0.99 |
| Comorbidities | 729 (4.8) | 51 (1.3) | <0.001 |
| Hypertension | 313 (2.1) | 28 (0.7) | <0.001 |
| Diabetes mellitus | 414 (2.7) | 46 (1.2) | <0.001 |
| Cancer | 617 (4.1) | 106 (2.7) | <0.001 |
| Chronic lung disease | 46 (0.3) | 2 (0.1) | 0.009 |
| Myocardial infarction | 30 (0.2) | 2 (0.1) | 0.076 |
| Heart failure | 126 (0.8) | 10 (0.3) | <0.001 |
| Cerebral infarction | 27 (0.2) | 3 (0.1) | 0.233 |
| Hyperlipidemia | 79 (0.5) | 8 (0.2) | 0.013 |
| Liver disease | 86 (0.6) | 5 (0.1) | 0.001 |
| Dementia | 69 (0.5) | 12 (0.3) | 0.262 |
| Gout |  |  |  |
| Telephone triage symptom |  |  | <0.001 |
| Common cold symptoms | 5831 (38.4) | 3069 (78.5) |  |
| Syncope | 1989 (13.1) | 60 (1.5) |  |
| Sore throat | 1255 (8.3) | 128 (3.3) |  |
| Headache | 980 (6.4) | 170 (4.3) |  |
| Diarrhea | 781 (5.1) | 75 (1.9) |  |
| Rash | 458 (3.0) | 40 (1.0) |  |
| Constipation | 397 (2.6) | 37 (0.9) |  |
| Allergic reaction | 314 (2.1) | 39 (1.0) |  |
| Fever (adult) | 263 (1.7) | 27 (0.7) |  |
| Ear ringing | 241 (1.6) | 14 (0.4) |  |
| Ankle to toe problem | 241 (1.6) | 8 (0.2) |  |
| Bruising | 221 (1.5) | 45 (1.2) |  |
| Lower extremity problem | 190 (1.2) | 5 (0.1) |  |
| Blood in stool | 176 (1.2) | 13 (0.3) |  |
| Laceration | 172 (1.1) | 12 (0.3) |  |
| Dyspnea | 125 (0.8) | 5 (0.1) |  |
| Upper extremity problem | 125 (0.8) | 4 (0.1) |  |
| Burn | 107 (0.7) | 12 (0.3) |  |
| Chest pain | 104 (0.7) | 4 (0.1) |  |
| Asthma | 80 (0.5) | 9 (0.2) |  |
| Back pain | 80 (0.5) | 2 (0.1) |  |
| Bleeding | 77 (0.5) | 5 (0.1) |  |
| Dizziness | 69 (0.5) | 2 (0.1) |  |
| Extremity/facial injury | 68 (0.4) | 2 (0.1) |  |
| Abdominal pain | 63 (0.4) | 54 (1.4) |  |
| Itching | 63 (0.4) | 2 (0.1) |  |
| Genital problems, Male | 60 (0.4) | 12 (0.3) |  |
| Wheezing | 50 (0.3) | 7 (0.2) |  |
| Mastalgia | 46 (0.3) | 8 (0.2) |  |
| Numbness, Sensory disturbance, Paralysis | 44 (0.3) | 1 (0.0) |  |
| Pain during urination | 43 (0.3) | 4 (0.1) |  |
| Hiccups | 42 (0.3) | 3 (0.1) |  |
| Palpitation | 39 (0.3) | 4 (0.1) |  |
| Fall | 37 (0.2) | 1 (0.0) |  |
| Abnormal urine color | 35 (0.2) | 3 (0.1) |  |
| Dysuria | 33 (0.2) | 1 (0.0) |  |
| Bites animal/human/insect/marine animal/snake | 30 (0.2) | 2 (0.1) |  |
| Heat stroke | 29 (0.2) | 1 (0.0) |  |
| Neck pain | 28 (0.2) | 0 (0.0) |  |
| Anxiety or fear | 27 (0.2) | 3 (0.1) |  |
| Wound healing and infection | 24 (0.2) | 0 (0.0) |  |
| Hyperventilation | 22 (0.1) | 3 (0.1) |  |
| Head injury | 17 (0.1) | 0 (0.0) |  |
| Heartburn | 13 (0.1) | 0 (0.0) |  |
| Neck injury | 13 (0.1) | 1 (0.0) |  |
| Vomiting or nausea | 12 (0.1) | 2 (0.1) |  |
| Hypertension | 12 (0.1) | 0 (0.0) |  |
| Disturbance of consciousness | 10 (0.1) | 2 (0.1) |  |
| Nasal injury | 10 (0.1) | 1 (0.0) |  |
| Eye injury | 8 (0.1) | 0 (0.0) |  |
| Hearing loss | 6 (0.0) | 0 (0.0) |  |
| Food poisoning | 5 (0.0) | 0 (0.0) |  |
| Vaginal bleeding | 4 (0.0) | 0 (0.0) |  |
| Penetrating injury | 4 (0.0) | 0 (0.0) |  |
| Trunk injury | 4 (0.0) | 0 (0.0) |  |
| Earache, Drainage | 3 (0.0) | 5 (0.1) |  |
| Dysarthria | 2 (0.0) | 0 (0.0) |  |
| Eye problems | 2 (0.0) | 0 (0.0) |  |
| Nasal problem | 2 (0.0) | 0 (0.0) |  |
| Seizure | 2 (0.0) | 0 (0.0) |  |
| Contact lens problems | 2 (0.0) | 1 (0.0) |  |
| Foreign body, Skin | 2 (0.0) | 0 (0.0) |  |
| Lumbago | 1 (0.0) | 1 (0.0) |  |
| Insomnia | 1 (0.0) | 0 (0.0) |  |
| Depression | 1 (0.0) | 0 (0.0) |  |
| First aid for trauma and burns | 1 (0.0) | 0 (0.0) |  |
| Swallowing of foreign body | 1 (0.0) | 0 (0.0) |  |
| Accidental ingestion of liquids | 1 (0.0) | 0 (0.0) |  |
| Foreign body, Eye | 1 (0.0) | 1 (0.0) |  |
| Foreign body, Nose | 1 (0.0) | 0 (0.0) |  |
| Fish bone in pharynx | 1 (0.0) | 0 (0.0) |  |
| Foreign body, Rectum | 1 (0.0) | 0 (0.0) |  |
| Foreign body, Vagina | 1 (0.0) | 0 (0.0) |  |
| Hypothermia | 1 (0.0) | 0 (0.0) |  |

| Table S3. Characteristics of patients with undertriage   \|  \| Patients with undertriage (n=298) \| \| --- \| --- \| \| Age (mean ± SD) \| 58.2 ± 23.9 \| \| Male (%) \| 164 (55.0) \| \| Triage color \|  \| \| Yellow \| 88 (29.5) \| \| Orange \| 210 (70.5) \| \| Comorbidities \|  \| \| Hypertension \| 43 (14.4) \| \| Diabetes mellitus \| 8 (2.7) \| \| Cancer \| 23 (7.7) \| \| Chronic lung disease \| 25 (8.4) \| \| Myocardial infarction \| 5 (1.7) \| \| Heart failure \| 2 (0.7) \| \| Cerebral infarction \| 5 (1.7) \| \| Hyperlipidemia \| 0 (0.0) \| \| Liver disease \| 3 (1.0) \| \| Dementia \| 14 (4.7) \| \| Gout \| 3 (1.0) \| \| Telephone triage symptom \|  \| \| Common cold symptoms \| 61 (20.5) \| \| Sore throat \| 39 (13.1) \| \| Syncope \| 34 (11.4) \| \| Diarrhea \| 22 (7.4) \| \| Allergic reaction \| 19 (6.4) \| \| Bruising \| 14 (4.7) \| \| Lower extremity problem \| 12 (4.0) \| \| Ear ringing \| 10 (3.4) \| \| Constipation \| 8 (2.7) \| \| Headache \| 7 (2.3) \| \| Dyspnea \| 6 (2.0) \| \| Fever (adult) \| 5 (1.7) \| \| Blood in stool \| 5 (1.7) \| \| Upper extremity problem \| 5 (1.7) \| \| Ankle to toe problem \| 5 (1.7) \| \| Rash \| 3 (1.0) \| \| Abdominal pain \| 3 (1.0) \| \| Wheezing \| 3 (1.0) \| \| Dizziness \| 3 (1.0) \| \| Hyperventilation \| 3 (1.0) \| \| Laceration \| 3 (1.0) \| \| Extremity/facial injury \| 3 (1.0) \| \| Heat stroke \| 3 (1.0) \| \| Back pain \| 3 (1.0) \| \| Genital problems, Male \| 2 (0.7) \| \| Itching \| 2 (0.7) \| \| Fall \| 2 (0.7) \| \| Wound healing and infection \| 2 (0.7) \| \| Lumbago \| 1 (0.3) \| \| Vomiting or nausea \| 1 (0.3) \| \| Pain during urination \| 1 (0.3) \| \| Abnormal urine color \| 1 (0.3) \| \| Asthma \| 1 (0.3) \| \| Anxiety or fear \| 1 (0.3) \| \| Nasal problem \| 1 (0.3) \| \| Disturbance of consciousness \| 1 (0.3) \| \| Bleeding \| 1 (0.3) \| \| Burn \| 1 (0.3) \| \| Chest pain \| 1 (0.3) \|   All categorical variables are presented as n (%). Continuous variables are shown as mean ± SD or median (interquartile range).  SD, standard deviation  Table S4. Comparison of patient characteristics between the training and test datasets | | | |
| --- | --- | --- | --- | --- | --- | --- | --- | --- | --- | --- | --- | --- | --- | --- | --- | --- | --- | --- | --- | --- | --- | --- | --- | --- | --- | --- | --- | --- | --- | --- | --- | --- | --- | --- | --- | --- | --- | --- | --- | --- | --- | --- | --- | --- | --- | --- | --- | --- | --- | --- | --- | --- | --- | --- | --- | --- | --- | --- | --- | --- | --- | --- | --- | --- | --- | --- | --- | --- | --- | --- | --- | --- | --- | --- | --- | --- | --- | --- | --- | --- | --- | --- | --- | --- | --- | --- | --- | --- | --- | --- | --- | --- | --- | --- | --- | --- | --- | --- | --- | --- | --- | --- | --- | --- | --- | --- | --- | --- | --- | --- | --- | --- | --- | --- | --- | --- | --- | --- | --- |
|  | Training set (n = 10,949) | Test set (n = 4,693) | *P-value* |
| Age (mean ± SD) | 39.4 ± 17.4 | 39.4 ±17.6 | 0.78 |
| Male (%) | 4716 (43.1) | 2040 (43.5) | 0.66 |
| Triage color | 7576 (69.2) | 3210 (68.4) | 0.34 |
| Yellow | 3373 (30.8) | 1483 (31.6) |  |
| Orange | 7576 (69.2) | 3210 (68.4) | 0.34 |
| Undertriage | 218 (2.0) | 98 (2.1) | 0.74 |
| Comorbidities |  |  |  |
| Hypertension | 507 (4.6) | 248 (5.3) | 0.09 |
| Diabetes mellitus | 230 (2.1) | 96 (2.0) | 0.87 |
| Cancer | 284 (2.6) | 146 (3.1) | 0.08 |
| Chronic lung disease | 438 (4.0) | 193 (4.1) | 0.78 |
| Myocardial infarction | 29 (0.3) | 18 (0.4) | 0.28 |
| Heart failure | 20 (0.2) | 11 (0.2) | 0.64 |
| Cerebral infarction | 85 (0.8) | 47 (1.0) | 0.19 |
| immune deficiency | 16 (0.1) | 11 (0.2) | 0.31 |
| Hyperlipidemia | 158 (1.4) | 78 (1.7) | 0.34 |
| Liver disease | 59 (0.5) | 21 (0.4) | 0.54 |
| Dementia | 66 (0.6) | 22 (0.5) | 0.36 |
| Gout | 55 (0.5) | 18 (0.4) | 0.38 |
| Telephone triage symptom |  |  | 0.47 |
| Common cold symptoms | 4074 (38.3) | 1764 (38.5) |  |
| Syncope | 1377 (13.0) | 613 (13.4) |  |
| Sore throat | 925 (8.7) | 331 (7.2) |  |
| Headache | 688 (6.5) | 292 (6.4) |  |
| Diarrhea | 550 (5.2) | 232 (5.1) |  |
| Rash | 315 (3.0) | 143 (3.1) |  |
| Constipation | 280 (2.6) | 118 (2.6) |  |
| Allergic reaction | 221 (2.1) | 93 (2.0) |  |
| Fever (adult) | 175 (1.6) | 88 (1.9) |  |
| Ear ringing | 171 (1.6) | 70 (1.5) |  |
| Ankle to toe problem | 161 (1.5) | 80 (1.7) |  |
| Bruising | 154 (1.4) | 67 (1.5) |  |
| Lower extremity problem | 137 (1.3) | 53 (1.2) |  |
| Laceration | 129 (1.2) | 44 (1.0) |  |
| Blood in stool | 112 (1.1) | 64 (1.4) |  |
| Upper extremity problem | 89 (0.8) | 36 (0.8) |  |
| Dyspnea | 83 (0.8) | 42 (0.9) |  |
| Burn | 76 (0.7) | 31 (0.7) |  |
| Chest pain | 71 (0.7) | 33 (0.7) |  |
| Back pain | 57 (0.5) | 23 (0.5) |  |
| Asthma | 53 (0.5) | 27 (0.6) |  |
| Bleeding | 52 (0.5) | 25 (0.5) |  |
| Abdominal pain | 48 (0.5) | 15 (0.3) |  |
| Genital problems, Male | 48 (0.5) | 12 (0.3) |  |
| Dizziness | 47 (0.4) | 22 (0.5) |  |
| Extremity/facial injury | 45 (0.4) | 23 (0.5) |  |
| Itching | 41 (0.4) | 22 (0.5) |  |
| Wheezing | 35 (0.3) | 15 (0.3) |  |
| Mastalgia | 34 (0.3) | 13 (0.3) |  |
| Numbness, Sensory disturbance, Paralysis | 30 (0.3) | 14 (0.3) |  |
| Fall | 28 (0.3) | 9 (0.2) |  |
| Pain during urination | 27 (0.3) | 16 (0.3) |  |
| Abnormal urine color | 27 (0.3) | 8 (0.2) |  |
| Palpitation | 27 (0.3) | 12 (0.3) |  |
| Hiccups | 24 (0.2) | 18 (0.4) |  |
| Anxiety or fear | 20 (0.2) | 7 (0.2) |  |
| Dysuria | 19 (0.2) | 14 (0.3) |  |
| Bites animal/human/insect/marine animal/snake | 19 (0.2) | 11 (0.2) |  |
| Neck pain | 18 (0.2) | 10 (0.2) |  |
| Heat stroke | 17 (0.2) | 12 (0.3) |  |
| Wound healing and infection | 15 (0.1) | 9 (0.2) |  |
| Hyperventilation | 14 (0.1) | 8 (0.2) |  |
| Head injury | 12 (0.1) | 5 (0.1) |  |
| Neck injury | 11 (0.1) | 2 (0.0) |  |
| Heartburn | 9 (0.1) | 4 (0.1) |  |
| Disturbance of consciousness | 8 (0.1) | 2 (0.0) |  |
| Nasal injury | 8 (0.1) | 2 (0.0) |  |
| Vomiting or nausea | 6 (0.1) | 6 (0.1) |  |
| Hypertension | 6 (0.1) | 6 (0.1) |  |
| Eye injury | 5 (0.0) | 3 (0.1) |  |
| Hearing loss | 4 (0.0) | 2 (0.0) |  |
| Penetrating injury | 4 (0.0) | 0 (0.0) |  |
| Earache, Drainage | 3 (0.0) | 0 (0.0) |  |
| Trunk injury | 3 (0.0) | 1 (0.0) |  |
| Nasal problem | 2 (0.0) | 0 (0.0) |  |
| Foreign body, Skin | 2 (0.0) | 0 (0.0) |  |
| Food poisoning | 2 (0.0) | 3 (0.1) |  |
| Dysarthria | 1 (0.0) | 1 (0.0) |  |
| Vaginal bleeding | 1 (0.0) | 3 (0.1) |  |
| Depression | 1 (0.0) | 0 (0.0) |  |
| Eye problems | 1 (0.0) | 1 (0.0) |  |
| First aid for trauma and burns | 1 (0.0) | 0 (0.0) |  |
| Seizure | 1 (0.0) | 1 (0.0) |  |
| Swallowing of foreign body | 1 (0.0) | 0 (0.0) |  |
| Foreign body, Eye | 1 (0.0) | 0 (0.0) |  |
| Foreign body, Nose | 1 (0.0) | 0 (0.0) |  |
| Fish bone in pharynx | 1 (0.0) | 0 (0.0) |  |
| Foreign body, Rectum | 1 (0.0) | 0 (0.0) |  |
| Hypothermia | 1 (0.0) | 0 (0.0) |  |
| Lumbago | 0 (0.0) | 1 (0.0) |  |
| Insomnia | 0 (0.0) | 1 (0.0) |  |
| Accidental ingestion of liquids | 0 (0.0) | 1 (0.0) |  |
| Contact lens problems | 0 (0.0) | 2 (0.0) |  |
| Foreign body, Vagina | 0 (0.0) | 1 (0.0) |  |

All categorical variables are presented as n (%). Continuous variables are shown as mean ± SD or median (interquartile range).

SD, standard deviation

Table S5. Lasso logistic regression for the undertriage

|  | β coefficient |
| --- | --- |
| Age | 0.66 |
| Triage color | 0.09 |
| Comorbidities |  |
| Diabetes mellitus | 0.44 |
| Cancer | 0.19 |
| Cerebral infarction | 0.25 |
| Dementia | 0.19 |
| Gout | 0.55 |
| Telephone triage symptom |  |
| Dyspnea | 0.76 |
| Syncope | 0.18 |
| Common cold symptoms | 0.39 |

Table S6. Comparison of patient characteristics on the training set before and after SMOTE-NC

|  | Training data before SMOTE-NC (n = 13,379) | Training data after SMOTE-NC (n = 26,332) | *p-value* |
| --- | --- | --- | --- |
| Age mean ± SD | 38.3 ± 16.6) | 48.05 ± 23.0) | <0.001 |
| Male (%) | 7674 (57.4) | 15,153 (57.5) | 0.729 |
| Triage color |  |  |  |
| Yellow |  |  |  |
| Orange | 9753 (72.9) | 18818 (71.5) | 0.003 |
| Undertriage | 213 (1.6) | 13166 (50.0) | <0.001 |
| Comorbidities |  |  |  |
| Hypertension | 573 (4.3) | 2636 (10.0) | <0.001 |
| Diabetes mellitus | 249 (1.9) | 977 (3.7) | <0.001 |
| Cancer | 311 (2.3) | 796 (3.0) | <0.001 |
| Chronic lung disease | 508 (3.8) | 508 (1.9) | <0.001 |
| Myocardial infarction | 33 (0.2) | 33 (0.1) | 0.007 |
| Heart failure | 24 (0.2) | 24 (0.1) | 0.025 |
| Cerebral infarction | 91 (0.7) | 273 (1.0) | 0.001 |
| Hyperlipidemia | 171 (1.3) | 171 (0.6) | <0.001 |
| Liver disease | 65 (0.5) | 65 (0.2) | <0.001 |
| Dementia | 61 (0.5) | 182 (0.7) | 0.006 |
| Gout | 58 (0.4) | 58 (0.2) | <0.001 |
| Telephone triage symptom |  |  | <0.001 |
| Common cold symptoms | 6190 (46.3) | 9570 (36.3) |  |
| Syncope | 1455 (10.9) | 3017 (11.5) |  |
| Sore throat | 980 (7.3) | 3300 (12.5) |  |
| Headache | 799 (6.0) | 1076 (4.1) |  |
| Diarrhea | 592 (4.4) | 1496 (5.7) |  |
| Rash | 343 (2.6) | 351 (1.3) |  |
| Constipation | 308 (2.3) | 419 (1.6) |  |
| Allergic reaction | 249 (1.9) | 1385 (5.3) |  |
| Fever (adult) | 213 (1.6) | 396 (1.5) |  |
| Ear ringing | 186 (1.4) | 344 (1.3) |  |
| Bruising | 184 (1.4) | 926 (3.5) |  |
| Ankle to toe problem | 183 (1.4) | 404 (1.5) |  |
| Lower extremity problem | 140 (1.0) | 699 (2.7) |  |
| Blood in stool | 133 (1.0) | 462 (1.8) |  |
| Laceration | 127 (0.9) | 138 (0.5) |  |
| Dyspnea | 97 (0.7) | 310 (1.2) |  |
| Upper extremity problem | 94 (0.7) | 263 (1.0) |  |
| Abdominal pain | 81 (0.6) | 144 (0.5) |  |
| Burn | 78 (0.6) | 78 (0.3) |  |
| Chest pain | 75 (0.6) | 131 (0.5) |  |
| Asthma | 65 (0.5) | 65 (0.2) |  |
| Bleeding | 60 (0.4) | 60 (0.2) |  |
| Back pain | 57 (0.4) | 137 (0.5) |  |
| Dizziness | 49 (0.4) | 87 (0.3) |  |
| Genital problems, Male | 47 (0.4) | 71 (0.3) |  |
| Itching | 47 (0.4) | 47 (0.2) |  |
| Extremity/facial injury | 45 (0.3) | 67 (0.3) |  |
| Wheezing | 40 (0.3) | 40 (0.2) |  |
| Numbness, Sensory disturbance, Paralysis | 36 (0.3) | 36 (0.1) |  |
| Hiccups | 33 (0.2) | 33 (0.1) |  |
| Palpitation | 31 (0.2) | 31 (0.1) |  |
| Pain during urination | 30 (0.2) | 30 (0.1) |  |
| Mastalgia | 30 (0.2) | 30 (0.1) |  |
| Fall | 29 (0.2) | 29 (0.1) |  |
| Abnormal urine color | 28 (0.2) | 53 (0.2) |  |
| Dysuria | 27 (0.2) | 27 (0.1) |  |
| Bites animal/human/insect/marine animal/snake | 22 (0.2) | 22 (0.1) |  |
| Neck pain | 20 (0.1) | 20 (0.1) |  |
| Anxiety or fear | 18 (0.1) | 35 (0.1) |  |
| Hyperventilation | 16 (0.1) | 16 (0.1) |  |
| Heat stroke | 16 (0.1) | 184 (0.7) |  |
| Wound healing and infection | 15 (0.1) | 88 (0.3) |  |
| Head injury | 15 (0.1) | 15 (0.1) |  |
| Disturbance of consciousness | 11 (0.1) | 53 (0.2) |  |
| Neck injury | 9 (0.1) | 9 (0.0) |  |
| Heartburn | 8 (0.1) | 8 (0.0) |  |
| Hypertension | 8 (0.1) | 8 (0.0) |  |
| Vomiting or nausea | 7 (0.1) | 7 (0.0) |  |
| Earache, Drainage | 7 (0.1) | 7 (0.0) |  |
| Nasal injury | 7 (0.1) | 7 (0.0) |  |
| Hearing loss | 6 (0.0) | 6 (0.0) |  |
| Eye injury | 5 (0.0) | 5 (0.0) |  |
| Vaginal bleeding | 3 (0.0) | 3 (0.0) |  |
| Penetrating injury | 3 (0.0) | 3 (0.0) |  |
| Trunk injury | 3 (0.0) | 3 (0.0) |  |
| Food poisoning | 3 (0.0) | 3 (0.0) |  |
| Nasal problem | 2 (0.0) | 54 (0.2) |  |
| Seizure | 2 (0.0) | 2 (0.0) |  |
| Contact lens problems | 2 (0.0) | 2 (0.0) |  |
| Dysarthria | 1 (0.0) | 1 (0.0) |  |
| Lumbago | 1 (0.0) | 11 (0.0) |  |
| Swallowing of foreign body | 1 (0.0) | 1 (0.0) |  |
| Accidental ingestion of liquids | 1 (0.0) | 1 (0.0) |  |
| Foreign body, Eye | 1 (0.0) | 1 (0.0) |  |
| Foreign body, Nose | 1 (0.0) | 1 (0.0) |  |
| Foreign body, Rectum | 1 (0.0) | 1 (0.0) |  |
| Foreign body, Vagina | 1 (0.0) | 1 (0.0) |  |
| Foreign body, Skin | 1 (0.0) | 1 (0.0) |  |
| Hypothermia | 1 (0.0) | 1 (0.0) |  |

Table S7. Comparison of patient characteristics between the training set after SMOTE-NC and test set

|  | Training data after SMOTE-NC (n = 26,332) | Test data (n = 5,735) | *p-value* |
| --- | --- | --- | --- |
| Age mean ± SD | 48.1 (23.0) | 38.6 (16.7) | <0.001 |
| Male (%) | 15153 (57.5) | 3268 (57.0) | 0.44 |
| Triage color |  |  |  |
| Yellow | 18818 (71.5) | 4152 (72.4) | 0.16 |
| Orange |  |  |  |
| Undertriage | 13166 (50.0) | 89 (1.6) | <0.001 |
| Comorbidities |  |  |  |
| Hypertension | 2636 (10.0) | 207 (3.6) | <0.001 |
| Diabetes mellitus | 977 (3.7) | 92 (1.6) | <0.001 |
| Cancer | 796 (3.0) | 149 (2.6) | 0.09 |
| Chronic lung disease | 508 (1.9) | 215 (3.7) | <0.001 |
| Myocardial infarction | 33 (0.1) | 15 (0.3) | 0.03 |
| Heart failure | 24 (0.1) | 8 (0.1) | 0.41 |
| Cerebral infarction | 273 (1.0) | 45 (0.8) | 0.09 |
| Hyperlipidemia | 171 (0.6) | 67 (1.2) | <0.001 |
| Liver disease | 65 (0.2) | 22 (0.4) | 0.10 |
| Dementia | 182 (0.7) | 30 (0.5) | 0.18 |
| Gout | 58 (0.2) | 23 (0.4) | 0.02 |
| Telephone triage symptom |  |  | <0.001 |
| Common cold symptoms | 9570 (36.3) | 2710 (47.3) |  |
| Syncope | 3017 (11.5) | 594 (10.4) |  |
| Sore throat | 3300 (12.5) | 403 (7.0) |  |
| Headache | 1076 (4.1) | 351 (6.1) |  |
| Diarrhea | 1496 (5.7) | 264 (4.6) |  |
| Rash | 351 (1.3) | 155 (2.7) |  |
| Constipation | 419 (1.6) | 126 (2.2) |  |
| Allergic reaction | 1385 (5.3) | 104 (1.8) |  |
| Bruising | 926 (3.5) | 82 (1.4) |  |
| Fever (adult) | 396 (1.5) | 77 (1.3) |  |
| Ear ringing | 344 (1.3) | 69 (1.2) |  |
| Ankle to toe problem | 404 (1.5) | 66 (1.2) |  |
| Laceration | 138 (0.5) | 57 (1.0) |  |
| Blood in stool | 462 (1.8) | 56 (1.0) |  |
| Lower extremity problem | 699 (2.7) | 55 (1.0) |  |
| Burn | 78 (0.3) | 41 (0.7) |  |
| Abdominal pain | 144 (0.5) | 36 (0.6) |  |
| Upper extremity problem | 263 (1.0) | 35 (0.6) |  |
| Dyspnea | 310 (1.2) | 33 (0.6) |  |
| Chest pain | 131 (0.5) | 33 (0.6) |  |
| Genital problems, Male | 71 (0.3) | 25 (0.4) |  |
| Extremity/facial injury | 67 (0.3) | 25 (0.4) |  |
| Back pain | 137 (0.5) | 25 (0.4) |  |
| Asthma | 65 (0.2) | 24 (0.4) |  |
| Mastalgia | 30 (0.1) | 24 (0.4) |  |
| Dizziness | 87 (0.3) | 22 (0.4) |  |
| Bleeding | 60 (0.2) | 22 (0.4) |  |
| Itching | 47 (0.2) | 18 (0.3) |  |
| Wheezing | 40 (0.2) | 17 (0.3) |  |
| Pain during urination | 30 (0.1) | 17 (0.3) |  |
| Heat stroke | 184 (0.7) | 14 (0.2) |  |
| Hiccups | 33 (0.1) | 12 (0.2) |  |
| Palpitation | 31 (0.1) | 12 (0.2) |  |
| Anxiety or fear | 35 (0.1) | 12 (0.2) |  |
| Abnormal urine color | 53 (0.2) | 10 (0.2) |  |
| Bites animal/human/insect/marine animal/snake | 22 (0.1) | 10 (0.2) |  |
| Numbness, Sensory disturbance, Paralysis | 36 (0.1) | 9 (0.2) |  |
| Hyperventilation | 16 (0.1) | 9 (0.2) |  |
| Fall | 29 (0.1) | 9 (0.2) |  |
| Wound healing and infection | 88 (0.3) | 9 (0.2) |  |
| Neck pain | 20 (0.1) | 8 (0.1) |  |
| Vomiting or nausea | 7 (0.0) | 7 (0.1) |  |
| Dysuria | 27 (0.1) | 7 (0.1) |  |
| Heartburn | 8 (0.0) | 5 (0.1) |  |
| Neck injury | 9 (0.0) | 5 (0.1) |  |
| Hypertension | 8 (0.0) | 4 (0.1) |  |
| Nasal injury | 7 (0.0) | 4 (0.1) |  |
| Eye injury | 5 (0.0) | 3 (0.1) |  |
| Eye problems | 0 (0.0) | 2 (0.0) |  |
| Head injury | 15 (0.1) | 2 (0.0) |  |
| Food poisoning | 3 (0.0) | 2 (0.0) |  |
| Dysarthria | 1 (0.0) | 1 (0.0) |  |
| Lumbago | 11 (0.0) | 1 (0.0) |  |
| Vaginal bleeding | 3 (0.0) | 1 (0.0) |  |
| Earache, Drainage | 7 (0.0) | 1 (0.0) |  |
| Insomnia | 0 (0.0) | 1 (0.0) |  |
| Depression | 0 (0.0) | 1 (0.0) |  |
| Disturbance of consciousness | 53 (0.2) | 1 (0.0) |  |
| Penetrating injury | 3 (0.0) | 1 (0.0) |  |
| First aid for trauma and burns | 0 (0.0) | 1 (0.0) |  |
| Trunk injury | 3 (0.0) | 1 (0.0) |  |
| Foreign body, Eye | 1 (0.0) | 1 (0.0) |  |
| Contact lens problems | 2 (0.0) | 1 (0.0) |  |
| Fish bone in pharynx | 0 (0.0) | 1 (0.0) |  |
| Foreign body, Skin | 1 (0.0) | 1 (0.0) |  |
| Hearing loss | 6 (0.0) | 0 (0.0) |  |
| Nasal problem | 54 (0.2) | 0 (0.0) |  |
| Seizure | 2 (0.0) | 0 (0.0) |  |
| Swallowing of foreign body | 1 (0.0) | 0 (0.0) |  |
| Accidental ingestion of liquids | 1 (0.0) | 0 (0.0) |  |
| Foreign body, Nose | 1 (0.0) | 0 (0.0) |  |
| Foreign body, Rectum | 1 (0.0) | 0 (0.0) |  |
| Foreign body, Vagina | 1 (0.0) | 0 (0.0) |  |
| Hypothermia | 1 (0.0) | 0 (0.0) |  |

Table S8. Results of sensitivity analyses.

| Classifiers | Sensitivity | Specificity | PPV | NPV | AUROC (95% CI) |
| --- | --- | --- | --- | --- | --- |
| Complete case analysis |  |  |  |  |  |
| Support vector machine | 50.5 | 51.8 | 2.1 | 98.1 | 0.52 (0.45–0.58) |
| Lasso regression | 76.3 | 69.0 | 4.9 | 99.3 | 0.78 (0.73-0.83) |
| Random forest | 68.8 | 76.2 | 5.7 | 99.2 | 0.80 (0.75–0.84) |
| Gradient-boosted decision tree | 71.0 | 73.3 | 5.2 | 99.2 | 0.78 (0.73–0.83) |
| Deep neural network | 72.0 | 74.2 | 5.5 | 99.2 | 0.79 (0.74–0.84) |
| Under-sampling method |  |  |  |  |  |
| Support vector machine | 53.9 | 71.4 | 2.9 | 99.0 | 0.62 (0.55–0.70) |
| Lasso regression | 70.8 | 73.8 | 4.1 | 99.4 | 0.79 (0.74-0.83) |
| Random forest | 71.9 | 77.9 | 4.9 | 99.4 | 0.81 (0.76–0.86) |
| Gradient-boosted decision tree | 70.8 | 75.7 | 4.4 | 99.4 | 0.80 (0.75–0.84) |
| Deep neural network | 77.5 | 69.7 | 3.9 | 99.5 | 0.78 (0.73–0.82) |
| Over-sampling method |  |  |  |  |  |
| Support vector machine | 65.2 | 66.1 | 2.9 | 99.2 | 0.69 (0.63–0.74) |
| Lasso regression | 71.9 | 70.2 | 3.7 | 99.4 | 0.72 (0.66-0.79) |
| Random forest | 70.8 | 73.5 | 4.0 | 99.4 | 0.74 (0.68–0.79) |
| Gradient-boosted decision tree | 68.5 | 67.8 | 3.2 | 99.3 | 0.73 (0.67–0.78) |
| Deep neural network | 57.3 | 80.2 | 4.4 | 99.2 | 0.71 (0.65–0.77) |
| Min-max normalization to age |  |  |  |  |  |
| Support vector machine | 50.5 | 51.8 | 2.1 | 98.1 | 0.52 (0.45–0.58) |
| Lasso regression | 76.3 | 69.2 | 4.9 | 99.3 | 0.78 (0.73-0.83) |
| Random forest | 77.4 | 67.5 | 4.7 | 99.3 | 0.79 (0.74–0.83) |
| Gradient-boosted decision tree | 71.0 | 73.5 | 5.3 | 99.2 | 0.78 (0.73–0.83) |
| Deep neural network | 67.7 | 75.9 | 5.5 | 99.1 | 0.79 (0.76–0.84) |
| AUROC, area under the receiver operating characteristic curve; CI, confidence interval; PPV, positive predictive value; NPV, negative predictive value | | | | | |

## Table S9. TRIPOD Checklist

| **Section/Topic** | **Item** | **D/V^*^** | **Checklist Item** | **Section/Paragraph number** |
| --- | --- | --- | --- | --- |
| Title | 1 | D;V | Identify the study as developing and/or validating a multivariable prediction model, the target population, and the outcome to be predicted. | Title page |
| Abstract | 2 | D;V | Provide a summary of objectives, study design, setting, participants, sample size, predictors, outcome, statistical analysis, results, and conclusions. | Abstract |
| Background and objectives | 3a | D;V | Explain the medical context (including whether diagnostic or prognostic) and rationale for developing or validating the multivariable prediction model, including references to existing models. | Introduction/#2 |
|  | 3b | D;V | Specify the objectives, including whether the study describes the development or validation of the model or both. | Introduction/#4 |
| Source of data | 4a | D;V | Describe the study design or source of data (e.g., randomized trial, cohort, or registry data), separately for the development and validation datasets, if applicable. | Methods, Study design, patient selection, and data handling / #1 |
|  | 4b | D;V | Specify the key study dates, including start of accrual; end of accrual; and, if applicable, end of follow-up. | Methods, Study design, patient selection, and data handling/#1, #3 |
| Participants | 5a | D;V | Specify key elements of the study setting (e.g., primary care, secondary care, general population) including number and location of centers. | Supplementary, Material and methods, Data sources |
|  | 5b | D;V | Describe eligibility criteria for participants. | Methods, Study design, patient selection, and data handling/#2 |
|  | 5c | D;V | Give details of treatments received, if relevant. | Methods, Risk factors and outcomes/#1 |
| Outcome | 6a | D;V | Clearly define the outcome that is predicted by the prediction model, including how and when assessed. | Methods, Risk factors and outcomes/#1 |
|  | 6b | D;V | Report any actions to blind assessment of the outcome to be predicted. | N/A because the data source is existing database |
| Predictors | 7a | D;V | Clearly define all predictors used in developing or validating the multivariable prediction model, including how and when they were measured. | Table 1 |
|  | 7b | D;V | Report any actions to blind assessment of predictors for the outcome and other predictors. | N/A because the data source is existing database |
| Sample size | 8 | D;V | Explain how the study size was arrived at. | N/A because the data source is existing database |
| Missing data | 9 | D;V | Describe how missing data were handled (e.g., complete case analysis, single imputation, multiple imputation) with details of any imputation method. | Methods, Risk factors and outcomes/#3 |
| Statistical analysis methods | 10a | D | Describe how predictors were handled in the analyses. | Methods, Risk factors and outcomes/#4 |
|  | 10b | D | Specify type of model, all model-building procedures (including any predictor selection), and method for internal validation. | Methods, Machine learning algorithms/#4, |
|  | 10c | V | For validation, describe how the predictions were calculated. | Methods, Risk factors and outcomes/#4 |
|  | 10d | D;V | Specify all measures used to assess model performance and, if relevant, to compare multiple models. | Methods, Risk factors and outcomes/#4 |
|  | 10e | V | Describe any model-updating (e.g., recalibration) arising from the validation, if done. | N/A because no updating was done |
| Risk groups | 11 | D;V | Provide details on how risk groups were created, if done. | N/A because no risk group was created |
| Development vs. validation | 12 | V | For validation, identify any differences from the development data in setting, eligibility criteria, outcome, and predictors. | Methods, Risk factors and outcomes/#4 |
| Participants | 13a | D;V | Describe the flow of participants through the study, including the number of participants with and without the outcome and, if applicable, a summary of the follow-up time. A diagram may be helpful. | Figure 1 |
|  | 13b | D;V | Describe the characteristics of the participants (basic demographics, clinical features, available predictors), including the number of participants with missing data for predictors and outcome. | Table 1, Supplementary, Tables S1-3 |
|  | 13c | V | For validation, show a comparison with the development data of the distribution of important variables (demographics, predictors and outcome). | Supplementary, Table S2 |
| Model development | 14a | D | Specify the number of participants and outcome events in each analysis. | Supplementary, Table S1 |
|  | 14b | D | If done, report the unadjusted association between each candidate predictor and outcome. | Supplementary, Table S4 |
| Model specification | 15a | D | Present the full prediction model to allow predictions for individuals (i.e., all regression coefficients, and model intercept or baseline survival at a given time point). | Results #2 |
|  | 15b | D | Explain how to use the prediction model. | Results #3 |
| Model performance | 16 | D;V | Report performance measures (with CIs) for the prediction model. | Results # |
| Model-updating | 17 | V | If done, report the results from any model-updating (i.e., model specification, model performance). | N/A because no updating was done |
| Limitations | 18 | D;V | Discuss any limitations of the study (e.g., nonrepresentative sample, few events per predictor, missing data). | Discussion, Strengths, and limitations/#1 |
| Interpretation | 19a | V | For validation, discuss the results with reference to performance in the development data, and any other validation data. | Discussion/#2 |
|  | 19b | D;V | Give an overall interpretation of the results, considering objectives, limitations, results from similar studies, and other relevant evidence. | Discussion/#1, #2 |
| Implications | 20 | D;V | Discuss the potential clinical use of the model and implications for future research. | Discussion/#4 |
| Supplementary information | 21 | D;V | Provide information about the availability of supplementary resources, such as study protocol, Web calculator, and datasets. | Described in the manuscript |
| Funding | 22 | D;V | Give the source of funding and the role of the funders for the present study. | Abstract, Funding |

*Items relevant only to the development of a prediction model are denoted by D, items relating solely to the validation of a prediction model are denoted by V, and items relating to both are denoted D;V.
